# Ultra-Barcoding Discovers a Cryptic Species in *Paris yunnanensis* (Melanthiaceae), a Medicinally Important Plant

**DOI:** 10.3389/fpls.2020.00411

**Published:** 2020-04-22

**Authors:** Yunheng Ji, Changkun Liu, Jin Yang, Lei Jin, Zhenyan Yang, Jun-Bo Yang

**Affiliations:** ^1^CAS Key Laboratory for Plant Diversity and Biogeography of East Asia, Kunming Institute of Botany, Chinese Academy of Sciences, Kunming, China; ^2^Yunnan Key Laboratory for Integrative Conservation of Plant Species with Extremely Small Populations, Kunming Institute of Botany, Chinese Academy of Sciences, Kunming, China; ^3^School of Life Sciences, Yunnan University, Kunming, China; ^4^School of Traditional Chinese Medicine, Guangdong Pharmaceutical University, Guangzhou, China; ^5^Germplasm Bank of Wild Species, Kunming Institute of Botany, Chinese Academy of Sciences, Kunming, China

**Keywords:** new species, plastomes, ribosomal DNA, species identification, DNA barcodes, *Paris liiana*, Melanthiaceae

## Abstract

Ultra-barcoding is a technique using whole plastomes and nuclear ribosomal DNA (nrDNA) sequences for plant species identification. *Paris yunnanensis* is a medicinal plant of great economic importance for the pharmaceutical industry. However, the alpha taxonomy of *P. yunnanensis* is still uncertain, hindering effective conservation and management of the germplasm. To resolve long-standing taxonomic disputes regarding this species, we newly generated the complete plastomes and nrDNA sequences from 22 *P. yunnanensis* accessions. Ultra-barcoding analyses suggest that *P. yunnanensis* as currently circumscribed is made up of two distinct genetic lineages, corresponding to the two phenotypes (“typical” and “high stem” form) identified early in our study. With distinct morphologies and distribution, the “high stem” form should be recognized as a previously unrecognized species; here it is described as a new species, *P. liiana* sp. nov. Moreover, the ultra-barcoding data do not support treatment of *P. yunnanensis* as a conspecific variety under *Paris polyphylla*. Our study represents a guiding practical application of ultra-barcoding for discovery of cryptic species in taxonomically challenging plant taxa. The findings highlight the great potential of ultra-barcoding as an effective tool for resolving perplexing problems in plant taxonomy.

## Introduction

DNA barcoding involves the standardized use of one or a few DNA regions for identification and discrimination of species (Hebert et al., 2003; Hollingsworth, 2011; Hollingsworth et al., 2016), as well as the discovery of cryptic or novel species (Hebert et al., 2003; Bell et al., 2012). Although the mitochondrial gene *cytochrome oxidase 1* (*COI*) performs well as a standard animal DNA barcode (Ward et al., 2005; Hajibabaei et al., 2006; Pons et al., 2006), reliable species discrimination based on standard DNA barcodes (i.e., *rbcL*, *matK*, *trnH-psbA*, and ITS) remains problematic in plants (Hollingsworth et al., 2009, 2016; Hollingsworth, 2011; Li et al., 2011; Coissac et al., 2016). With the advent of next-generation DNA sequencing (NGS) technologies, the concept of DNA barcoding for plant species has been extended from one or several sequence loci to large amounts of genomic data (Li et al., 2015; Coissac et al., 2016; Hollingsworth et al., 2016). Complete plastid genomes (plastomes) and entire nuclear ribosomal DNA (nrDNA) sequences harbor many more sequence variations, making them far more sensitive and effective than standard DNA barcodes, especially among very closely related taxa (Nock et al., 2011; Kane et al., 2012; Ruhsam et al., 2015; Ji et al., 2019a; Zhu et al., 2019; Li et al., 2020). The extension of standard DNA barcodes to whole plastomes and nrDNA sequences has been referred to as “ultra-barcoding” (Kane et al., 2012). However, practical application of this technique for discovery of cryptic or novel species in taxonomically difficult plant taxa is still absent from literature.

*Paris yunnanensis* Franch. (Melanthiaceae), a perennial rhizomatous herb distributed in southwestern China and northern Myanmar (Li, 1998), has great economic value. Dried rhizome of this plant, bearing the pharmaceutical name “*Rhizoma Paridis*,” is a traditional medicine in China with hemostatic, anti-inflammatory, analgesic, antipyretic, and other therapeutic properties (China Pharmacopoeia Commission, 2015). Phytochemical investigations revealed steroidal saponins as the main components responsible for the bioactivities of this plant (Yang Z. Y. et al., 2019). There are about 70 commercial drugs and health products that use *Rhizoma Paridis* as raw materials, including “*Yunnan Baiyao*,” a famous Chinese medicine, and “*Gongxuening Capsule*,” a gynecological hemostatic based on extractions of *Rhizoma Paridis*. The value of these pharmaceutical products is estimated to be more than 10 billion CNY (∼1.5 billion USD) per year (Huang et al., 2012).

**FIGURE 1:**
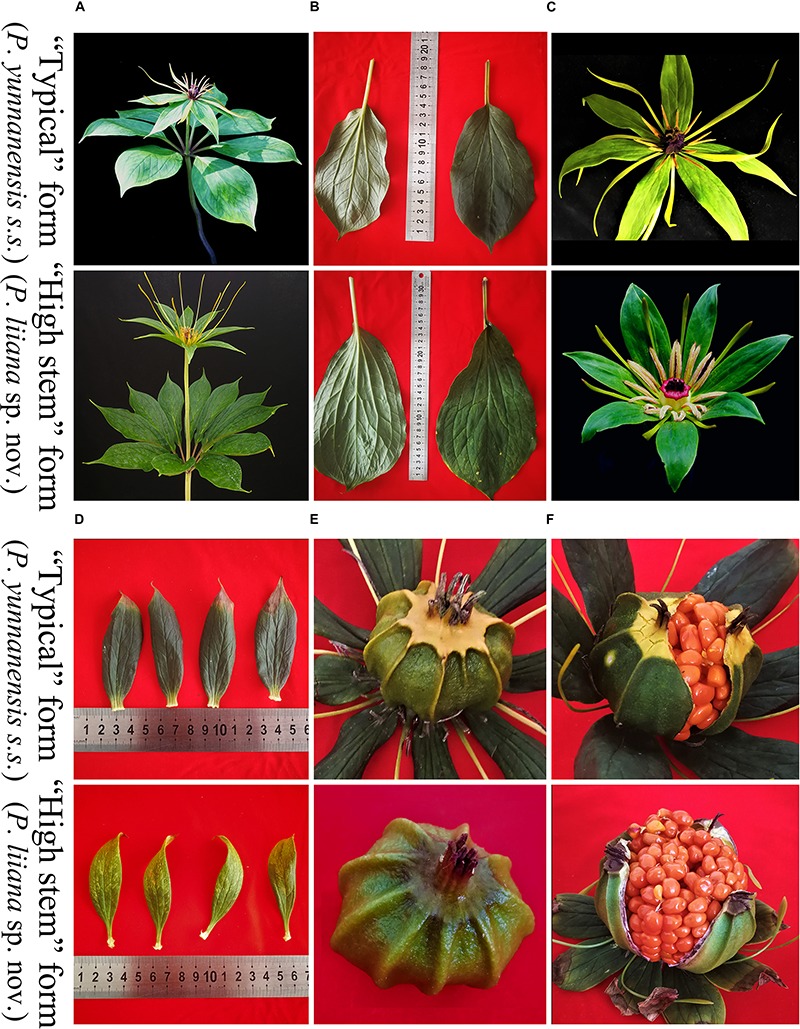
Comparison of morphological features between “typical” *Paris yunnanensis* (*P. yunnanensis s.s.*) and “high stem” form (*P. liiana* sp. nov.). **(A)** aerial shoot. **(B)** leaf shape and size. **(C)** flower. **(D)** sepals. **(E)** young fruit. **(F)** mature fruit.

Although *Paris* is morphologically distinct from other angiosperm genera, the rhizome, leaf, flower, stamen, ovary, fruit, and seed morphologies, which have been widely used for classification, are highly divergent among species (Hara, 1969; Takhtajan, 1983; Li, 1998; Ji et al., 2006, 2019b). Since the description of *P. yunnanensis* by Franchet (1888), its taxonomic rank has been in dispute. Handel-Mazzetti (1936) proposed that the morphologies of *P. yunnanensis* are largely homologous to those of *Paris polyphylla*, and thus reduced it to a conspecific variety (*P. polyphylla* var. *yunnanensis*) of the latter species; this treatment was followed by Hara (1969) and Li (1998). However, Takhtajan (1983) argued that *P. yunnanensis* is morphologically different from *P. polyphylla* and should be treated as a separate species. We detected a number of morphological differences among *P. yunnanensis* accessions during the early stage of our study, based on which we identified two phenotypes (“typical” form and “high stem” form, Figure 1). The morphological variation within *P. yunnanensis* suggests that the taxonomic delimitation of this economically important plant needs to be re-assessed. Given the great economic importance of *P. yunnanensis*, satisfactory resolution of these taxonomic issues will be conductive to exploration and protection of its germplasm.

Genome skimming, involving a relatively low coverage shotgun sequencing of genomic DNA, is an efficient and cost-effective approach to recover highly repetitive genome components such as nrDNA or organelle genomes (Straub et al., 2012). The genome skimming approach using NGS can recover plastomes, nrDNA clusters and sometimes even the complete nuclear genome at relatively low sequencing depth, and these sequence data can be both backwards-compatible with the standard plant barcodes, and forward-compatible with whole genome sequencing (Straub et al., 2012; Coissac et al., 2016; Hollingsworth et al., 2016). Because of the significant advantages, this approach has great promise for extending the concept of DNA barcoding from one or a few DNA regions to genomes (Hollingsworth et al., 2016). Recently, genome skimming has been employed to genomic data for species discrimination in several taxonomically challenging plant groups, for instance, *Theobroma* (Kane et al., 2012), *Araucaria* (Ruhsam et al., 2015), *Diospyros* (Turner et al., 2016), and *Panax* (Ji et al., 2019a).

In this study, we sampled 22 *P. yunnanensis* individuals, representing the two phenotypes identified, and generated complete plastomes and nrDNA sequences for these individuals using a genome skimming approach. Based on ultra-barcoding analyses, we aimed to elucidate (1) whether *P. yunnanensis* is related closely enough to *P. polyphylla* to warrant taxonomic treatment as conspecific varieties, and (2) whether the two phenotypes within *P. yunnanensis* represent distinct taxa.

## Materials and Methods

### Plant Materials and Low-Coverage Shotgun Sequencing of Genomes

A total of 22 individuals of *P. yunnanensis* as currently circumscribed (16 accessions of “typical” form and 6 accessions of “high stem” form) were collected from the wild according to records of herbarium specimens, approximately covering the geographic range of the species (Table 1). The vouchers were identified by Dr. Yunheng Ji and deposited at the herbarium of the Kunming Institute of Botany, Chinese Academy of Sciences (KUN).

**TABLE 1:** Voucher information and GenBank accessions for the 22 *Paris yunnanensis* accessions.

Voucher number	Locality	Latitude (N)	Longitude (E)	Elevation (m)	nrDNA accession number	Plastome accession number
**“Typical” form (*Paris yunnanensis s.s.*)**						
HYL02	Chuxiong, Yunnan	25° 00′ 27.36″	101° 26′ 54.43″	2,300	MN647562	MN175241
JYH2016403	Huize, Yunnan	25° 58′ 37.14″	103° 31′ 43.67″	2,100	MN647568	MN686112
JYH2016413	Weishan, Yunnan	25° 13′ 53.49″	100° 21′ 25.01″	2,284	MN647569	MN662251
JYH2016424	Huidong, Sichuan	26° 06′ 14.67″	104° 10′ 57.57″	2,350	MN647570	MN175244
JYH2016433	Waingmaw, Myanmar	25° 11′ 50.23″	97° 45′ 18.15″	1893	MN647571	MN686113
JYH2016489	Huili, Sichuan	26° 33′ 41.36″	102° 08′ 19.34″	2,150	MN647573	MN175240
JYH2016498	Ninglang, Yunnan	27° 17′ 26.58″	101° 02′ 12.18″	24,50	MN647574	MN175253
JYH2016503	Dongchuan, Yunnan	26° 19′ 06.4″	102° 58′ 09.31″	2,100	MN647576	MN175245
JYH2016504	Luquan, Yunnan	24° 58′ 36.78″	102° 28′ 35.27″	2,450	MN647577	MN686103
JYH2016507	Yongping, Yunnan	25° 24′ 33.33″	99° 18′ 58.83″	1,396	MN647578	MN175246
JYH2016515	Muli, Sichuan	27° 59′ 06.47″	100° 03′ 28.41″	2,630	MN647579	MN686104
JYH2016516	Lanping, Yunnan	26° 25′ 31.30″	99° 23′ 15.74″	2,646	MN647580	MN686105
JYH2016517	Shangri-la	27° 10′ 23.14″	100° 02′ 46.79″	2,265	MN647581	MN686106
JYH2017039	Mianning, Sichuan	28° 35′ 14.63″	102° 08′ 23.74″	1,840	MN647582	MN686107
JYH2017041	Shimian, Sichuan	29° 25′ 17.33″	102° 08′ 13.71″	1,700	MN647584	MN686109
JYH2017046	Yanyuan, Sichuan	27° 33′ 31.46″	101° 01′ 55.61 ″	2,484	MN647583	MN686108
**“High stem’′ form (*P. liiana sp. nov.*)**						
JYH2016457	Yuanyang, Yunnan	24° 43′ 53.76″	104° 21′ 06.01″	1599	MN647572	MN175247
HYL06	Qiubei, Yunnan	24° 03′ 55.45″	104° 10′ 57.57″	1,530	MN647563	MN175249
HYL07	Xinping, Yunnan	24° 00′ 03.82″	102° 00′ 41.04″	1,936	MN647564	MN175254
HYL08	Jinghong, Yunnan	22° 06′ 32.77″	100° 59’25.38″	980	MN647565	MN175248
HYL12	Xichou, Yunnan	23° 31’12.69″	105° 06′ 29.74″	1,107	MN647566	MN686111
HYL13	Mojiang, Yunnan	23° 33′ 23.99″	101° 42′ 13.38″	1,926	MN647567	MN175242

Genomic DNA was extracted from ∼20 mg silica-dried leaf tissues, using the cetyltrimethylammonium bromide (CTAB) method (Doyle and Doyle, 1987). Approximately 5 μg purified genomic DNA was sheared to fragments of 300–500 bp by sonication. Paired-end libraries with an average insert size of 350 bp were prepared using a TruSeq DNA Sample Prep Kit (Illumina, Inc., United States), according to the manufacturer’s instructions. The libraries were paired-end sequenced on the Illumina HiSeq 2000 platform. Raw reads were filtered to remove adaptors and low-quality reads using the NGS QC Toolkit (Patel and Jain, 2012), setting the cutoff value for percentage read length to 80 and Phred quality score to 30.

### Recovery and Annotation of Plastomes

High-quality reads were assembled to generate complete plastomes with GetOrganelle pipeline developed by Jin et al. (2018). The plastome sequence of *P. yunnanensis* (GenBank accession: MN125587) was used as a reference for plastome assembly. All of the plastid-like reads were assembled into contigs by SPAdes v3.10.1 (Bankevich et al., 2012) with the *k*-mer defined as 75, 85, 95, and 105. A customized python script (Jin et al., 2018), which uses BLAST and a built-in library to search the plastid-like contig, was employed to connect verified contigs into plastomes in Bowtie 2 (Langmead and Salzberg, 2012), with its default parameters.

The assembled plastomes were annotated using the Dual Organellar Genome Annotator database (Wyman et al., 2004). Start and stop codons and intron/exon boundaries for protein-coding genes were checked manually. Annotated tRNA genes were further verified using tRNAscan-SE 1.21 (Schattner et al., 2005) with default parameters. Gene content and arrangement of *P. yunnanensis* plastomes were visualized and compared using MUMmer 3.0 (Kurtz et al., 2004). Boundaries of the large single copy (LSC), inverted repeat (IR), and small single copy (SSC) regions in each plastome were compared using Geneious v10.2.3 (Kearse et al., 2012).

### Recovery of rDNA Sequences

Before the assembly of nrDNA clusters, all plastid-like reads were excluded from the Illumina data. The complete nrDNA sequence (including 26S, 18S, and 5.8S ribosomal RNA genes and ITS regions) of *P. yunnanensis* (MN174873) was used as a reference. Contigs mapping to reference nrDNA sequences were assembled using the processes described above. Nuclear ribosomal RNA genes and their boundaries with ITS regions were annotated and defined by comparison with the reference sequence using Geneious v10.2.3 (Kearse et al., 2012).

### Data Analysis

The efficiency of the complete plastomes and nrDNA sequences for species identification were investigated using tree-based methods. Based on the tree topologies, species-level monophyly of *P. yunnanensis* as currently circumscribed and its relationships with congeneric species were examined. In addition to the 22 *P. yunnanensis* plastomes and nrDNA sequences newly sequenced in this study (Table 1), 31 plastomes and nrDNA sequences determined from our previous studies (Huang et al., 2016; Ji et al., 2019a; Yang L. F. et al., 2019) and representing species in the genus *Paris* were included in the phylogenetic analyses. Plastome and nrDNA sequences were respectively aligned using the program MAFFT (Katoh and Standley, 2013) with manual adjustment where necessary. Alignment of sequences are deposited in the online database Treebase^1^.

Phylogenetic analysis of each dataset was performed using maximum likelihood (ML) and Bayesian inference (BI). The complete plastome (MN125577) and nrDNA (MN174897) sequences of *Trillium tschonoskii* were used as the outgroup to root the plastome and nuclear trees, respectively. Conflict between plastid and nuclear datasets was statistically tested using the incongruence length difference (ILD) test (Farris et al., 1994) implemented in PAUP^∗^ 4.0b10 (Swofford, 2002) for 1,000 replicates.

The best-fit substitution model for plastomes (GTR + G) and nrDNA (GTR + G + I) was determined using MODELTEST 3.7 (Posada and Crandall, 1998) with the Akaike information criterion (Posada and Buckley, 2004). ML analyses were performed in the software RAxML-HPC BlackBox v8.1.24 (Stamatakis, 2006). The best-scoring ML tree for each dataset was generated with 1,000 replicates to provide bootstrap percentage (BP) support values. BI analyses were performed using MrBayes v3.2 (Ronquist and Huelsenbeck, 2003). Two independent Markov Chain Monte Carlo (MCMC) simulations were run with 1,000,000 generations, sampling every 100 generations. An initial 25% of the sampled trees were discarded as burn-in. Posterior probability (PP) values were computed from the remaining trees. Stationarity was considered to be reached when the average standard deviation of the split frequencies was <0.01.

## Results

### Illumina Sequencing

Illumina sequencing generated between 9,448,962 and 25,342,424 paired-end clean reads per sample. Of those, 158,172–1,067,241 and 8,299–22,466 reads were mapped to the reference plastome and ribosomal DNA sequences, respectively (Supplementary Table S1). *De novo* assembly based on these data covered the entire plastome and nrDNA for all samples, with average coverage ranging from 44,917 to 1,011 and 211,637 to 572,407 times, respectively. The sequences newly generated in this study were deposited in NCBI GenBank, and their accession numbers are shown in Table 1.

### Phylogenies Based on nrDNA Sequences

**FIGURE 2:**
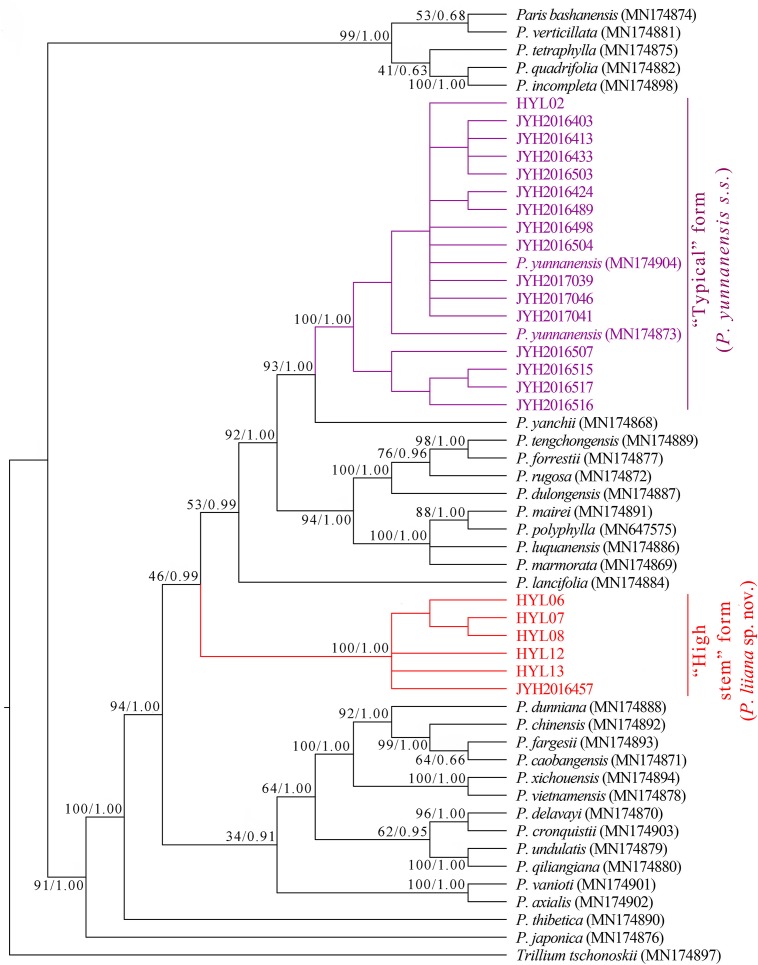
Phylogenetic tree reconstructed via maximum-likelihood (ML) and Bayesian inference (BI) analyses of nuclear ribosomal DNA (nrDNA) sequences. Numbers above branches indicate likelihood bootstrap percentages (BP) and Bayesian posterior probabilities (PP).

Assembly of nrDNA sequences entirely covered the 18S rDNA, ITS1, 5.8S rDNA, ITS2, and 26S rDNA clusters. Sequence lengths for the “typical” *P. yunnanensis* (16 accessions) and “high stem” phenotype (6 accessions) were 5,856 and 5,857 bp, respectively. Phylogenetic trees based on maximum likelihood (ML) and Bayesian inference (BI) analyses had a very similar topology overall, but exhibited minor differences within interior nodes. Both ML and BI analyses failed to recover all *P. yunnanensis* accessions as a monophyletic lineage, instead grouping them into two phylogenetically disparate clades (Figure 2). The first clade consisted of all “high stem” accessions while the second clade included all “typical” *P. yunnanensis* accessions. The monophyly of both clades received full branch support (BP = 100, PP = 1), and the clades were separated from each other by *Paris yanchii*, *Paris lancifolia* (≡ *P. polyphylla* var. *stenophylla*), and the clade comprising *Paris tengchongensis*, *Paris forrestii*, *Paris rugosa*, *Paris mairei*, *P. polyphylla*, *Paris luquanensis*, and *Paris marmorata*. Moreover, the nrDNA phylogenies indicated that *P. polyphylla* is sister to *P. mairei* (BP = 100, BI = 1) and closely related to *P. luquanensis* and *P. marmorata* (BP = 100, BI = 1). However, not only *P. yunnanensis* accessions but also *Paris chinensis* (≡ *P. polyphylla* var. *chinensis*) and *P. lancifolia*, once treated as conspecific varieties of *P. polyphylla* (Hara, 1969; Li, 1998), were phylogenetically disparate from *P. polyphylla* in both ML and BI trees (Figure 2).

### Plastome Phylogenies

**FIGURE 3:**
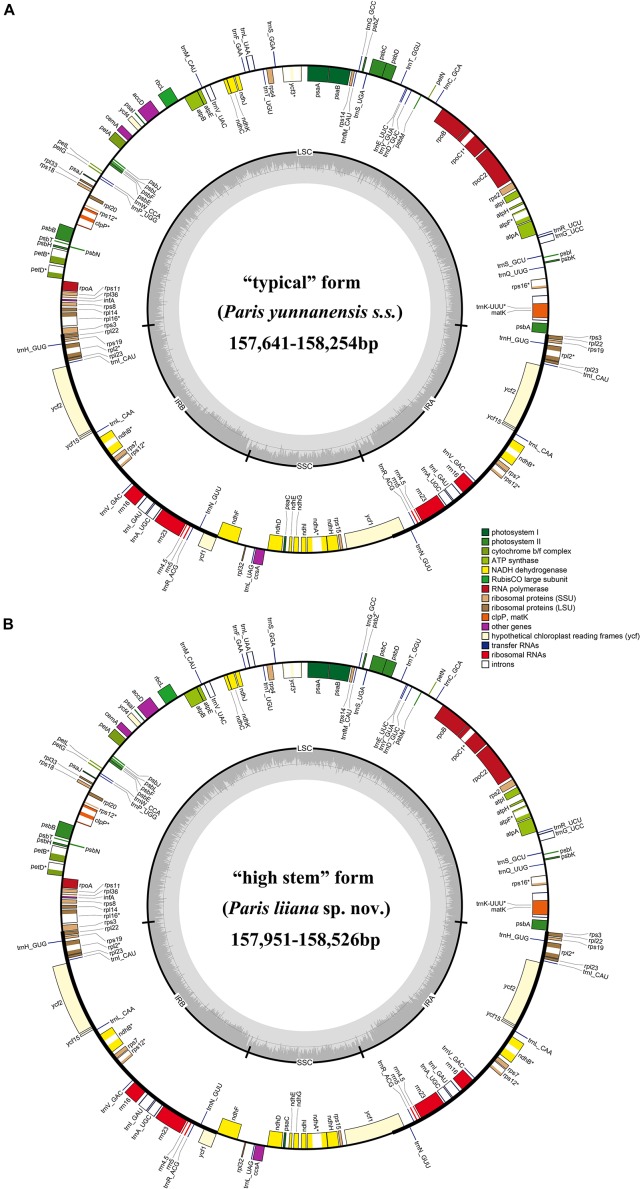
The plastome map of “typical” *Paris yunnanensis* (*P. yunnanensis s.s.*) accessions **(A)** and “high stem” form (*P. liiana* sp. nov) accessions **(B)**.

In this study, 22 *P. yunnanensis* plastomes were recovered, using the genome skimming approach. The plastome size of “typical” *P. yunnanensis* and “high stem” accessions varied from 157,641 to 158,254 bp and 157,951 to 158,526 bp, which possessed the typical quadripartite structure of flowering plants, consisting of a LSC, a SSC, and a pair of IRs (Figure 3). All plastomes contained the same 114 unique genes, including 80 protein-coding genes, 30 tRNA genes, and four rRNA genes (Supplementary Table S2). Several internal stop codons in coding regions of the *cemA* gene identified it as a pseudogene in all newly generated plastomes.

**FIGURE 4:**
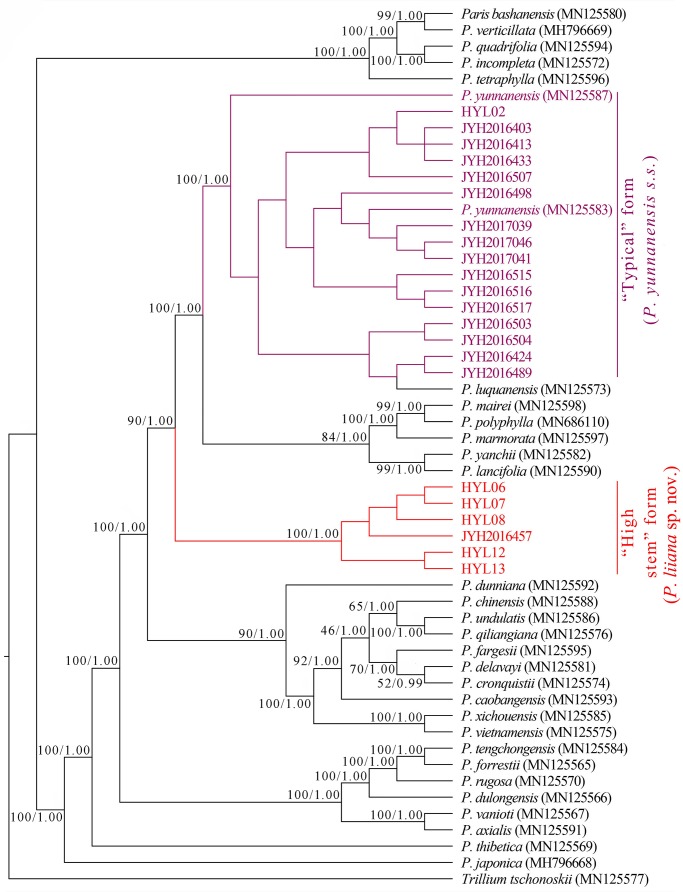
Phylogenetic tree reconstructed via maximum-likelihood (ML) and Bayesian inference (BI) analyses of complete plastomes. Numbers above branches indicate likelihood bootstrap percentages (BP) and Bayesian posterior probabilities (PP).

The incongruence length difference (ILD) test revealed strong discordance between plastome and nrDNA datasets (*p* < 0.001). Although both ML and BI analyses similarly grouped “high stem” and “typical” *P. yunnanensis* plastomes into two phylogenetically independent clades, the relationships of these two groups with congeneric species differed greatly from those revealed by nrDNA phylogenies. Since *P. luquanensis* was nested into “typical” *P. yunnanensis* accessions, both ML and BI analyses failed to resolve the latter as monophyletic (Figure 4). In addition, plastome phylogenies did not recover the sister relationships between “typical” *P. yunnanensis* accessions and *P. yanchii*, as well as between “high stem” accessions and *P. lancifolia*. Instead, *P. yanchii* and *P. lancifolia* formed a well-supported clade (BP = 99, PP = 1) sister to the clade consisting of *P. mairei*, *P. marmorata*, and *P. polyphylla* (BP = 84, PP = 1). Similar to nrDNA phylogenies, *P. chinensis*, *P. lancifolia*, *P. polyphylla*, and *P. yunnanensis* accessions were resolved as phylogenetically disparate in both tree topologies (Figure 4).

## Discussion

*Paris yunnanensis* is a medicinal plant with great economic importance to the pharmaceutical industry. In this study, we aimed to resolve long-standing taxonomic disputes regarding this species using ultra-barcoding technique. The complete plastomes and nuclear ribosomal DNA regions from 22 *Paris yunnanensis* accessions were newly generated to investigate the species-level monophyly of the plant and its relationships with the congeneric species. Our data not only allowed recognition of a cryptic species in *P. yunnanensis*, but also led us to resolve long-standing controversies regarding the taxonomic status of this species. This study represents a guiding practical application of ultra-barcoding technique for discovery of cryptic or novel species. The findings highlight the great potential of ultra-barcoding as an effective tool for resolving perplexing problems in taxonomically difficult plant taxa, and have implications for the conservation and management of *P. yunnanensis* germplasm.

### Putative Hybridization

Similar to previous studies (Ji et al., 2006, 2019b), we found that nrDNA and plastome phylogenies were largely incongruent in *Paris*. With respect to the target species of this study, the cytonuclear incongruence primarily involved the non-monophyly of “typical” *P. yunnanensis* accessions in plastome trees. Notably, cytonuclear discordance is a commonly investigated phenomenon in plant phylogenetics (Rieseberg and Soltis, 1991), which can be attributed to incomplete sorting of cytoplasmic polymorphisms or “chloroplast capture” resulting from hybridization (Wendel and Doyle, 1998). Ji et al. (2006) proposed that natural hybridization between some sympatric *Paris* species is feasible if the pollination mechanisms are compatible. In addition, observation of morphological intermediates between *P. yunnanensis* and *P. luquanensis* suggests that natural hybridization may occur between these two species (Ji et al., 2006).

Plastome tree topologies indicated that the non-monophyly of “typical” *P. yunnanensis* accessions results from clustering of *P. luquanensis* with *P. yunnanensis* accessions collected from northern Yunnan and southwestern Sichuan. Within these regions, *P. yunnanensis* is sympatric with *P. luquanensis.* Therefore, the non-monophyly of “typical” *P. yunnanensis* plastomes may have been caused by chloroplast capture, with the plastome from *P. yunnanensis* being introgressed into the nuclear background of *P. luquanensis* by hybridization (Rieseberg and Soltis, 1991; Rieseberg and Wendel, 1993). This assumption can be further tested through analyzing multiple loci of nuclear genes and sampling populations of both species.

### Evidence for a Cryptic Species Within *Paris yunnanensis*

The plasticity of morphological characteristics and lack of taxonomically robust characters among *Paris* species have made the taxonomy of this genus historically difficult to reconstruct, especially for Chinese and Himalayan species (Franchet, 1888; Hara, 1969; Takhtajan, 1983; Li, 1998). Despite the great commercial value of *P. yunnanensis* to the pharmaceutical industry, the alpha taxonomy of this plant is still uncertain. In this study, we used complete plastomes and nrDNA sequences as ultra-barcodes to assess the species-level monophyly of *P. yunnanensis* as currently circumscribed. Our data failed to group all accessions into a single and monophyletic clade, but resolved them as two phylogenetically disparate and well-supported clades corresponding to the two phenotypes identified in *P. yunnanensis*. This suggests that both “typical” and “high stem” forms represent two evolutionarily distinct lineages.

**FIGURE 5:**
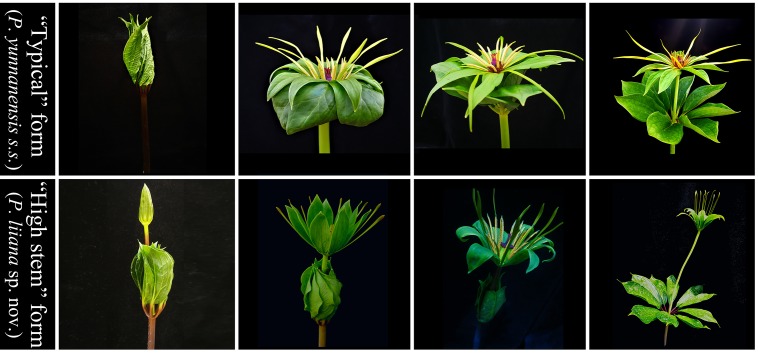
Comparison of the development of aerial shoot between “typical” *Paris yunnanensis* (*P. yunnanensis s.s.*) and “high stem” form (*P. liiana* sp. nov.).

**FIGURE 6:**
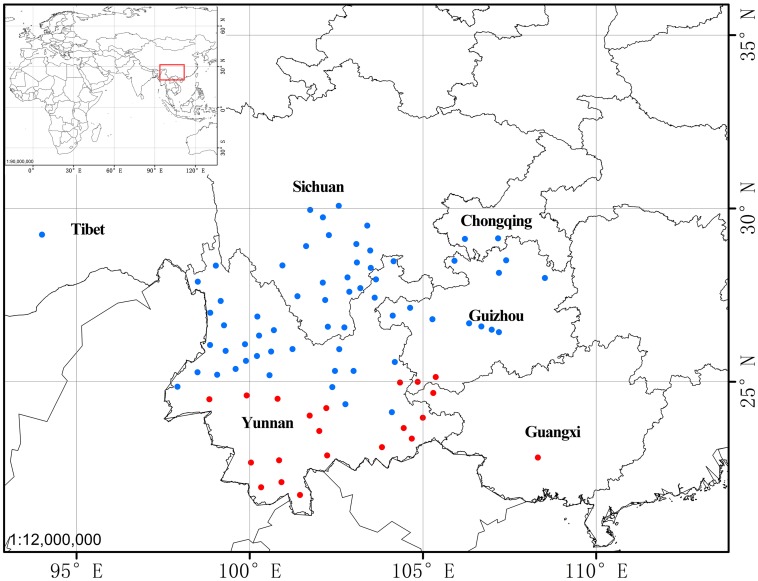
The distribution of *Paris yunnanensis s.s.* (“typical” form, blue cycle) and *P. liiana* sp. nov. (“high stem” form, red cycle).

The “high stem” form shows significant morphological differences from “typical” *P. yunnanensis*, which include plant height, leaf-blade shape, length, and width, sepal shape, petal color and width, and color of fruit at maturity (Figure 1 and Table 2). Interestingly, their aerial shoots also exhibit distinct growth patterns (Figure 5). Specifically, opening of flowers in “high stem” form is usually 5–15 days earlier than the leaf unfolding, when the pedicels extend out 10–30 cm above stem apex. On the contrast, flowering and leaf expansion synchronize in “typical” *P. yunnanensis*, whose pedicels do not obviously elongate until the full expansion of leaves. In addition, the two phenotypes possess distinct distribution ranges. The “high stem” populations occur in southern Yunnan, western Guangxi, and southwestern Guizhou, whereas “typical” *P. yunnanensis* (*P. yunnanensis s.s.*) is mainly distributed in central, northern, northwestern, and western Yunnan, southwestern Sichuan, and southeastern Tibet (Figure 6). There is little overlap between their respective distribution ranges. This evidence justifies the “high stem” form being recognized as a distinct taxon. Moreover, the phylogenetic relationships of the “high stem” form with related, well-defined, congeneric species suggest that recognition of it as a distinct species is appropriate.

**TABLE 2:** Morphological character differences between *Paris yunnanensis s.s.* and *P. liiana* sp. nov.

Character	“Typic” form (*Paris yunnanensis s.s.*)	“High stem” form (*P. liiana* sp. nov.)
Plant height	40–100 cm	80–200 cm
Leaf shape	Oblong	Elliptic, oblong-obovate
Leaf length/width	8–15 cm/3–7 cm	20–30 cm/8–15 cm
Sepal shape	Lanceolate	Oblong or obovate-oblong
Sepal length/width	4–7 cm/1.5–3 cm	5–12 cm/2.5–5 cm
Petals	Linear, 3–5 mm wide	Filiform-linear, 2–3 mm wide at the tip
Capsule	Yellow at the top	Dark red or brown at the top

The nrDNA and plastome tree topologies both indicated that *P. yunnanensis s.s.* (≡ *P. polyphylla* var. *yunnanensis*), *P. chinensis* (≡ *P. polyphylla* var. *chinensis*), and *P. lancifolia* (≡ *P. polyphylla* var. *stenophylla*) are genetically distinct from *P. polyphylla*. The ultra-barcoding data provide no support for treating these four taxa as conspecific varieties (Handel-Mazzetti, 1936; Hara, 1969; Li, 1998), but justify that they should be recognized as distinct species. It is notable that our sampling of *P. chinensis* and *P. lancifolia* (one individual per species) might be limiting for molecular study. Further study of their species-level monophyly by sampling multiple accessions per species is warranted.

With more variable characters than standard DNA barcodes, genomic data have been recommended as next-generation DNA barcodes for plants (Kane and Cronk, 2008; Nock et al., 2011; Kane et al., 2012; Ruhsam et al., 2015; Hollingsworth et al., 2016) and utilization of these extended barcodes in plant species identification is referred to as ultra-barcoding (Kane et al., 2012) or “plant barcoding 2.0” (Hollingsworth et al., 2016). However, the efficiency of ultra-barcodes for the discovery of cryptic and novel species has seldom been evaluated (Kane and Cronk, 2008; Kane et al., 2012; Hollingsworth et al., 2016). The practical application of ultra-barcodes in this study not only allowed recognition of a cryptic species in *P. yunnanensis*, but also led us to infer possible hybridization between *P. luquanensis* and “typical” *P. yunnanensis* (*P. yunnanensis s.s.*). Therefore, the ultra-barcoding approach has great promise for discovery of novel taxa, and offers significant advantages in interpreting possible hybridization events and identifying hybrids.

### Implications for the Management of *Paris yunnanensis* Germplasm Resources

Germplasm resources are the genetic material basis for plant breeding and crop improvement (Nass et al., 2012). Proper circumscription of a species and identification of germplasm diversity is a critical prerequisite to conservation and management efforts. We propose a narrow species delimitation for *P. yunnanensis*, based on successful distinction of *P. yunnanensis s.s.* from the cryptic species using complete plastome and nrDNA sequences as ultra-barcodes. In addition, both datasets possess high levels of infraspecific sequence variation in *P. yunnanensis s.s.* Thus, ultra-barcoding could be an effective tool for identifying *P. yunnanensis s.s.* and for investigating its germplasm diversity.

As we discussed above, cytonuclear discordance observed in *P. yunnanensis s.s.* implies that natural hybridization may occur between this plant and its sympatric congeneric species, *P. luquanensis*. From the perspective of germplasm conservation and management, great attention should be paid to the protection of “genetically genuine” individuals and populations. Ultra-barcoding may help exclude possible hybrids for construction of a core germplasm resource. Based on this, we could search for elite germplasm that is highly productive and contains high levels of steroidal saponins for breeding needs. Given that distant hybridization can either result in parental advantages or create heterosis (Cicin, 1954; Whitney et al., 2010), it will not only extend the germplasm resources of *P. yunnanensis s.s.*, but can also improve the performance of the outcrossing offspring. Therefore, hybrids are indispensable complements to the core germplasm resources. Ultra-barcoding will serve as a useful tool for genotyping hybrid germplasm and interpreting parentage. Elucidating these issues will improve future cross-breeding research.

### Taxonomic Treatment

**FIGURE 7:**
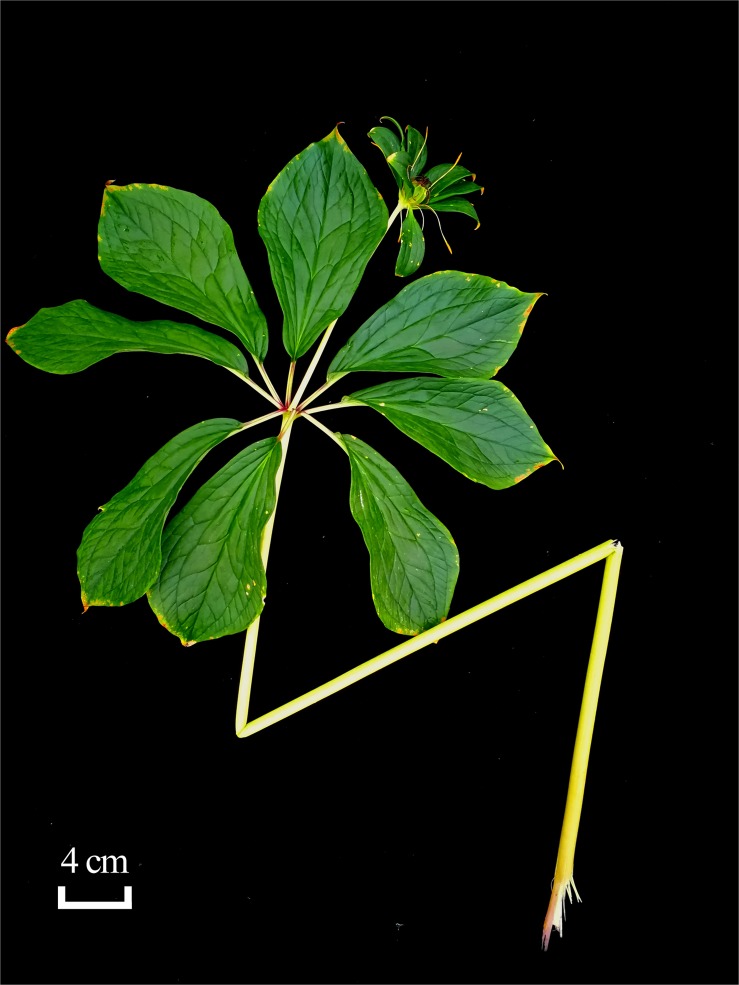
Holotype of *Paris liiana* sp. nov.

*Paris liiana* Y. H. Ji sp. nov. (Figures 1, 5, 7).

Type: China. Yunnan: Yuanyang County, Xiaoxinjie, 24° 43′ 53.76″ N, 104° 21′ 06.01″ E, 1599 m, 7 August 2016, *Y. H. Ji 2016457* (holotype, KUN!); Qiubei County, 24° 03′ 55.45″ N, 104° 10′ 57.57″ E, 1530 m, 12 July 2016, *Y. L. Huang 006* (paratype, KUN!).

*Paris liiana* can be distinguished from *P. yunnanensis* by its elliptic or oblong-obovate leaf blade, 20–30 cm × 8–15 cm, oblong or obovate-oblong sepals, petals filiform to linear, distally slightly widened to 2–3 mm, and capsule dark red or brown at the top.

Perennial herbs with cylindrical, oblique or horizontal rhizomes, yellowish brown outside, and white inside, 3.0–7.0 cm in diameter, 5.0–20.0 cm long, bearing a bud at the top, roots up to 30.0 cm long. Stem erect, cylindrical, purplish red or green, 50.0–150.0 cm tall. Leaves 5–12 in an apical whorl, green; petiole light green, 8.0–3.0 cm long; leaf blades elliptic or oblong-obovate, apex acute, 20–30 cm × 8–15 cm oblong, 6.0–19.0 × 3.5–9.5 cm; two pairs of lateral veins, basally developed. Flower solitary and terminal, basic merosity 5–10. Peduncle green or light purple, 25.0–50.0 cm; sepals 5–10, oblong or obovate-oblong, green, *ca.* 5–12 × 2.5–5 cm 3.5–8.6 × 1.6–2.2 cm; petals 5–10, filiform-linear, green at low portion, greenish yellow at upper portion, distally slightly widened to 2–3 mm, shorter or slightly longer than sepals. Stamens 2 × petal number, filament greenish yellow, 3.0–6.0 mm, anthers golden yellow, dehiscing by a lateral slit, 1.5–4.0 cm long. Ovary pale green at base, purplish red apically, with 5–10 slight ridges, carpels 5–10, unilocular with parietal placenta; style 4.0–5.0 mm, with an enlarged base, purplish red, stigmas 5–10-lobed, dark brown. Capsule dehiscent, subglobose, green, dark red or brown at the top. Seeds numerous, with a red and juicy sarcotesta.

Additional specimens examined: China. Guangxi: Longlin, 01 Jun. 1957, *Liang CF and Wu DL 32471* (IBSC); Nanning, 07 Jul. 1973, *Huang XC 5833* (GXMG). Guizhou: Anlong, 27° 44′ 34.7″ N, 98° 36′ 17.1″ E, 1800 m, 09 Jun. 1960, *Guizhou Expedition 3167* (KUN); loc. eodem, 09 Jun. 1960, *Zhang ZS and Zhang YT 4155* (PE); Xingyi, 20 Jul. 1960, *Zhang ZS and Zhang YT 6408* (PE). Yunnan: Eshan, 1350 m, 11 Jul. 1989, *Yuxi Expedition 89-516* (KUN); loc. eodem, 1300 m, 29 Apr. 1988, *Eshan Expedition 88-101* (KUN); Guangnan, 05 Jan. 2016, *Guangnan Expedition 5326270519* (IMDY); loc. eodem, 20 Jan. 2016, *Guangnan Expedition 5326270534* (IMDY); Jingdong, 21 Oct. 1956, *Qiu BY 52963* (KUN); loc. eodem, 28 Apr. 1959, *Xu SG 5049* (KUN); loc. eodem, 06 Dec. 1939, *Li MG 2263* (KUN); Jinghong, Sept. 1936, *Wang CW 78693* (PE); loc. eodem, May 1984, *Tao GD 44099* (HITBC); Menghai, May 1936, *Wang CW 74278* (PE); loc. eodem, 14 Jun. 2012, *Menghai Census 5328220509* (IMDY); loc. eodem, 24 Apr. 2012, *Menghai Census 5328220065* (IMDY); loc. eodem, 03 Oct. 1959, *Cai XT 59-10459* (KUN); loc. eodem, 1650 m, 17 Jun. 1960, *Yunnan Tropic Expedition 60-11693* (KUN); Mengla, 10 Nov. 1959, *Pei SJ 59-11386* (KUN); Lancang, 20 Aug. 2015, *Yang YP yi-173-1* (KUN); Longling, 17 May 2015, *Longling Census 530523150517045LY* (IMDY); Lüchun, 16 Oct. 1973, *Tao DD 856* (KUN); Luoping, 27 May 1989, *Hongshui River Expedition 1722* (KUN); loc. eodem, 2480 m, 28 May 1989, *Hongshui River Expedition 1922* (KUN); Fengqing, 18 Jun. 1938, *Yu TT 16348* (PE); Pingbian, 05 Oct. 1939, *Wang CW 82319* (PE); Simao, 29 May 2012, *Simao Census 5308020534* (IMDY); loc. eodem, 14 Jun. 2012, *Simao Census 5308020705* (IMDY); loc. eodem, 17 May 2012, *Simao Census 5308020404* (IMDY); Xichou, 2200 m, 24 Sept. 1947, *Feng GM 11993* (KUN); Xinping, 03 Jun. 2012, *Xinping Census 5304270419* (IMDY); Yanshan, 1250–1320 m, 09 Oct. 1939, *Wang QW 84252* (KUN); Yuanjiang, 08 Jun. 2012, *Yuanjiang Census 5304280573* (IMDY).

Etymology: The species is named in honor of Prof. Heng Li, who carried out the most recent and comprehensive taxonomic revision on the genus *Paris*.

Distribution: Guangxi, southwestern Guizhou, and southern Yunnan, China (Figure 6).

Habitat: Evergreen broad-leaved forests dominated by *Castanopsis*, *Lithocarpus*, *Quercus*, and *Schima* species at 1200–2200 elevation.

Phenology: Flowering April–May, fruiting June–December.

Conservation status: The species is commonly harvested as medicinal herb by local people. We estimate that its population size has been reduced by at least 50% over the past 10 years. According to the IUCN (2012) red list categories and criteria, *P. liinana* should be assessment as vulnerable status (VU A1d).

## Data Availability Statement

The datasets Generated for this study can be found in NCBI GenBank database, and the accession number of each sequence are showed in Table 1. Alignment of sequences are deposited in the online database Treebase (http://purl.org/phylo/treebase/phylows/study/TB2:S25503).

## Author Contributions

YJ and J-BY designed the research. CL, JY, LJ, ZY, and J-BY collected and analyzed the data. YJ wrote the manuscript. J-BY revised the manuscript.

## Conflict of Interest

The authors declare that the research was conducted in the absence of any commercial or financial relationships that could be construed as a potential conflict of interest.

## Footnotes

1 http://purl.org/phylo/treebase/phylows/study/TB2:S25503
