# Supplementary material for: Ultra-Barcoding Discovers a Cryptic Species in Paris yunnanensis (Melanthiaceae), a Medicinally Important Plant
Source: Front Plant Sci. 2020 Apr 22;11:411. doi: 10.3389/fpls.2020.00411 (PMC7189017; doi:10.3389/fpls.2020.00411)
Supplement: Supplementary file 1 [file Table_1.DOC]

Table S1. Summary of Illumina sequencing for 22 newly sequenced plastomes.

| Accession | No. of total reads | plastome | | | rDNA | | |
| --- | --- | --- | --- | --- | --- | --- | --- |
|  |  | No. of mapped reads | size of plastome (bp) | coverage (×) | No. of mapped reads | size of rDNA (bp) | coverage (×) |
| HYL02 | 25,342,424 | 475,303 | 157,802 | 450.598 | 16,211 | 5,856 | 413.405 |
| HYL06 | 22,668,038 | 334,091 | 158,252 | 316.726 | 12,290 | 5,857 | 313.414 |
| HYL07 | 23,321,320 | 383,654 | 158,128 | 363.713 | 10,312 | 5,857 | 262.972 |
| HYL08 | 24,623,282 | 294,049 | 158,526 | 278.765 | 15,150 | 5,857 | 386.348 |
| HYL12 | 25,964,476 | 372,493 | 157,951 | 353.132 | 16,248 | 5,857 | 414.349 |
| HYL13 | 22,953,960 | 627,031 | 158,138 | 594.440 | 9,641 | 5,857 | 245.860 |
| JYH2016403 | 23,175,774 | 1,067,241 | 157,724 | 1011.769 | 8,299 | 5,856 | 211.637 |
| JYH2016413 | 25,388,550 | 47,380 | 157,641 | 44.917 | 13,156 | 5,856 | 335.498 |
| JYH2016424 | 15,190,150 | 523,542 | 157,753 | 496.330 | 11,493 | 5,856 | 293.089 |
| JYH2016433 | 20,647,760 | 593,447 | 157,857 | 562.601 | 11,174 | 5,856 | 284.954 |
| JYH2016457 | 9,448,962 | 543,370 | 158,036 | 515.127 | 12,346 | 5,857 | 314.842 |
| JYH2016489 | 12,378,214 | 493,003 | 158,254 | 467.378 | 19,332 | 5,856 | 492.996 |
| JYH2016498 | 17,534,062 | 295,532 | 158,112 | 280.171 | 18,019 | 5,856 | 459.512 |
| JYH2016503 | 12,466,238 | 85,380 | 157,740 | 80.942 | 22,446 | 5,856 | 572.407 |
| JYH2016504 | 11,904,864 | 170,157 | 157,741 | 161.313 | 16,672 | 5,856 | 425.162 |
| JYH2016507 | 23,154,696 | 159,493 | 158,061 | 151.203 | 13,111 | 5,856 | 334.351 |
| JYH2016515 | 20,030,140 | 247,662 | 158,233 | 234.789 | 12,901 | 5,856 | 328.995 |
| JYH2016516 | 15,346,644 | 381,769 | 157,815 | 361.926 | 9,915 | 5,856 | 252.848 |
| JYH2016517 | 23,355,284 | 315,668 | 158,172 | 299.261 | 15,928 | 5,856 | 406.188 |
| JYH2017039 | 15,279,942 | 307,332 | 158,188 | 291.358 | 11,964 | 5,856 | 305.100 |
| JYH2017041 | 16,799,962 | 573,533 | 158,137 | 543.723 | 11,721 | 5,856 | 298.903 |
| JYH2017046 | 17,053,050 | 297,682 | 158,026 | 282.209 | 13,296 | 5,856 | 339.068 |
