# Supplementary material for: Ultra-Barcoding Discovers a Cryptic Species in Paris yunnanensis (Melanthiaceae), a Medicinally Important Plant
Source: Front Plant Sci. 2020 Apr 22;11:411. doi: 10.3389/fpls.2020.00411 (PMC7189017; doi:10.3389/fpls.2020.00411)
Supplement: Supplementary file 2 [file Table_2.DOCX]

Table S2. Gene content of 22 newly sequenced plastomes.

| Category of Genes | Group of gene | Name of gene |
| --- | --- | --- |
| Self-replication | Ribosomal RNA genes | *rrn*4.5×2*,rrn*5×2*,rrn*16×2*, rrn*23×2 |
|  | Transfer RNA genes | *trn*C_GCA*, trn*D_GUC*, trn*E_UUC*, trn*F_GAA*, trn*G_GCC*, trn*G_UCC**, trn*H_GUG×2*, trn*K_UUU**, trn*L_UAA**, trn*L_UAG*, trn*M_CAU*, trn*P_UGG*, trn*Q_UUG*, trn*R_UCU*, trn*S_GCU*, trn*S_GGA*, trn*S_UGA*, trn*T_UGU*, trn*T_GGU*, trn*V_UAC**, trn*Y_GUA*, trn*W_CCA*, trn*fM_CAU*, trn*A_UGC*×2*, trn*I_CAU*×*2*, trn*I_GAU*×2*, trn*L_CAA×2*, trn*N_GUU×2*, trn*R_ACG×2*, trn*V_GAC×2 |
|  | Ribosomal protein  (small subunit) | *rps*2*, rps*3*, rps*4*, rps*7×2*, rps*8*, rps*11*, rps*12**×2*, rps*14*, rps*15*, rps*16**, rps*18*, rps*19×2 |
|  | Ribosomal protein  (large subunit) | *rpl*2*×2*, rpl*14*, rpl*16**, rpl*20*, rpl*22×2*, rpl*23×2*, rpl*32*, rpl*33*, rpl*36 |
|  | RNA polymerase | *rpo*A*, rpo*B*, rpo*C1**, rpo*C2 |
|  | Translational initiation factor | *inf*A |
| Genes for photosynthesis | Subunits of photosystem I | *psa*A*, psa*B*, psa*C*, psa*I*，psa*J*, ycf*3***, ycf*4 |
|  | Subunits of photosystem II | *psb*A*, psb*B*, psb*C*, psb*D*, psb*E*, psb*F*, psb*H*, psb*I*, psb*J*, psb*K*, psb*L*, psb*M*, psb*N*, psb*T*, psb*Z |
|  | Subunits of cytochrome | *pet*A*, pet*B**, pet*D**, pet*G*, pet*L*, pet*N |
|  | Subunits of ATP synthase | *atp*A*, atp*B*, atp*E*, atp*F**, atp*H*, atp*I |
|  | Large subunit of Rubisco | *rbc*L |
|  | Subunits of NADH  Dehydrogenase | *ndh*A**, ndh*B*×2*, ndh*C*, ndh*D*, ndh*E*, ndh*F*, ndh*G*, ndh*H*, ndh*I*, ndh*J*, ndh*K |
| Other genes | Maturase | *mat*K |
|  | Envelope membrane protein | *cem*A^#^ |
|  | Subunit of acetyl-CoA | *acc*D |
|  | Synthesis gene | *ccs*A |
|  | ATP-dependent protease | *clp*P**** |
|  | Component of TIC complex | *ycf*1 |
| Genes of unknown function | Conserved open reading frames | *ycf*2×2*, ycf*15^#^×2 |

×2: Two gene copies in IR regions; *: With one intron; **: With two introns; #: Pseudogene.
